# Analysis and Identification of Bioactive Compounds of Cannabinoids in Silico for Inhibition of SARS-CoV-2 and SARS-CoV

**DOI:** 10.3390/biom12121729

**Published:** 2022-11-22

**Authors:** Chenxiao Chen, Hao Liang, Yanchun Deng, Xiushi Yang, Xiaoming Li, Chunsheng Hou

**Affiliations:** 1 Institute of Bast Fiber Crops and Center of Southern Economic Crops, Chinese Academy of Agricultural Sciences, Changsha 410205, China; 2 National Engineering Research Center for Vegetables, Institute of Vegetable Science, Beijing Academy of Agriculture and Forestry Sciences, Beijing 100097, China; 3 Key Laboratory of Urban Agriculture (North China), Ministry of Agriculture and Rural Affairs, Beijing 100097, China; 4 Bioengineering Research Center, Institute of Advanced Technology, Guangzhou 510000, China

**Keywords:** SARS-CoV-2, CBNA, luteolin, natural product, in silico

## Abstract

Despite the approval of multiple vaccinations in different countries, the majority of the world’s population remains unvaccinated due to discrepancies in vaccine distribution and limited production capacity. The SARS-CoV-2 RBD-ACE2 complex (receptor binding domain that binds to ACE2) could be a suitable target for the development of a vaccine or an inhibitor. Various natural products have been used against SARS-CoV-2. Here, we docked 42 active cannabinoids to the active site of the SARS-CoV-2 and SARS-CoV complex of RBD-ACE2. To ensure the flexibility and stability of the complex produced after docking, the top three ligand molecules with the best overall binding energies were further analyzed through molecular dynamic simulation (MDS). Then, we used the webserver Swissadme program and binding free energy to calculate and estimate the MMPBSA and ADME characteristics. Our results showed that luteolin, CBGVA, and CBNA were the top three molecules that interact with the SARS-CoV-2 RBD-ACE2 complex, while luteolin, stigmasterol, and CBNA had the strongest contact with that SARS-CoV. Our findings show that luteolin may be a potential inhibitor of infections caused by coronavirus-like pathogens such as COVID-19, although further in vivo and in vitro research is required.

## 1. Introduction

The ensuing outbreak of coronavirus disease 2019 (COVID-19) has now spread across the world, killing over six million people [1]. During viral infection, the spike glycoprotein (S) has been experimentally reported to be disassembled into S1 and S2 subunits [2]. The receptor binding domain (RBD) of ACE2 interacts directly with the peptidase domain [3]. Based on a recent study, the RBD is recognized by the peptidase domain of ACE2 via polar residues, which offers new insights into SARS-CoV-2 recognition and infection [4].

The three-dimensional structures of the SARS-CoV-2 spike (S) protein and ACE2 permitted the discovery of regions in the peptidase domain of ACE2 that are necessary for viral spike binding [5]. SARS-CoV and SARS-CoV-2 spike proteins also bind ACE2, which is highly expressed in type I and II alveolar cells in the lungs [6,7]. In the case of SARS-CoV, the spike protein’s S1 domain enables ACE2 receptor binding, but the S2 domain is a part that is linked with membranes and probably goes through post-binding trans-conformational alterations that enable membrane fusion. The amino acid residues 318 to 510 have been identified as the viral receptor binding domain (RBD) in S1 [8]. SARS-CoV-2 also found remarkable parallels between SARS-CoV and SARS-CoV-2 infection, including similar choices of entry receptors since the key amino acid residues necessary for ACE2 binding were retained [9]. Due to their critical involvement in SARS-CoV-2 or SARS-CoV life cycle, these two target areas have been intensively docked to build or identify structure-based effective medicines for COVID-19 [10].

A lethal virus known as COVID-19 is presently spreading around the world. Most of the world’s population remains unvaccinated as COVID-19 develops, despite the approval of multiple vaccinations in numerous countries due to differences in vaccine distribution and constrained production capacity. Scientists are investigating COVID-19 prevention strategies that employ both chemical and natural substances. Natural bioactive compounds are currently being screened using molecular docking to assess their affinity for molecular targets of COVID-19 in silico, with the benefit that natural products are free from harmful or adverse effects [11]. Several natural substances, including bee product alkaloids, terpenoids, marine products, and other natural products, have been confirmed as potential inhibitors against COVID-19 for targeting M^pro^, RdRp and Nsp of SARS-CoV-2 [12,13,14,15]. In addition, several natural products have been found that can block SARS-CoV-2 cell entry, including luteolin and CBDA [16,17]. Cannabis has recently been proposed as a potential COVID-19 inhibitor based on the bioactive substances it contains, such as CBD and THC [18]. *Cannabis sativa* L. has long been valued as a food, dietary fiber, nutritious oil, and medical source throughout Europe and Africa, especially in traditional Chinese medicine. The phenolic chemicals and terpenes found in *C. sativa* have been proven to have wound-healing, antioxidant, antibacterial, antiviral, anti-inflammatory, cardioprotective, and neurological effects [19]. Although there have been reports of cannabis inhibiting RdRP, spike protein and M^pro^, as well as other SARS-CoV-2 enzymes [20,21,22,23,24], little is known about the inhibition of the RBD-ACE2 complex of SARS-CoV-2. Additionally, the majority of these analyses have been applied to M^pro^ from SARS-CoV-2 [25,26,27].

Here, we employed molecular docking to test the binding affinity of 42 selected bioactive compounds, including terpenes and flavonoids, as inhibitors against the complex of RBD-ACE2 of SARS-CoV-2 or SARS-CoV. Luteolin and CBNA were not only safe but also had high absorption and bioavailability. These findings indicated these small-molecular cannabis inhibitors that block the entry of SARS-CoV-2 or SARS-CoV, suggesting that cannabis could be used as a development candidate inhibitor for preventing coronavirus-like.

## 2. Material and methods

### 2.1. Protein and Ligand Preparation

Protein Data Bank (https://www.rcsb.org/, accessed on 16 March 2022) provided the three-dimensional crystal structures of the SARS-CoV-2-RBD (PDB ID: 6M17) complex and SARS-CoV-RBD (PDB ID:3R4D) with 2.9 Å and 3.1 Å resolution, respectively [5,28]. To identify cannabinoids of the potential antiviral activities, we obtained bioactive molecules of cannabis from PubMed (https://pubchem.ncbi.nlm.nih.gov/, accessed on 18 March 2022) and traditional Chinese medicine system pharmacology (TCMSP) (https://tcmsp-e.com/tcmsp.php, accessed on 18 March 2022) as well as other reports with 2D structures in SDF file format [29,30,31,32]. We obtain a total of 42 non-repeat bioactive compounds to use as ligands for further investigation.

### 2.2. In Silico Molecular Docking

The ligand of molecules was molecularly docked to the active site of RBD-ACE2 of SARS-CoV-2 or SARS-CoV using the AutoDock vina docking software according to the normal approach [33]. The crystal structure of the protein was created before the docking procedure. The protein structure was modified to include hydrogen atoms, and all ionizable residues were set to their default protonation at pH 7.4.

The ligands were made in the same manner, and the energy was minimized using Avogadro software [33]. During docking, the receptor was tightly held, whereas the ligands were allowed to flex during refinement. Additionally, the interactions and binding energies between ligands and macromolecules were assessed computationally [34]. In the BIO-VIA Discovery Studio, a visualizer was employed to see the interactions between ligands and proteins.

### 2.3. Molecular Dynamics Simulations

To ensure flexibility and stability of the complex produced after docking, the top three ligand molecules with the best overall binding energies were put through 50 ns of molecular dynamic simulation (MDS) via version 2022.1 of GROningen MAchine for Chemical Simulations (GROMACS) [35]. In MDS, the topologies of the protein and ligand were initially collected using the ‘pdb2gmx’ script and the ACPYPE server, respectively, and then linked together to generate a conformation, which was then optimized using the amber ff14sb force field [36]. The ACPYPE service received the molecules’ PDB coordinates and converted them into topologies that could be used by GROMACS and other programs. Then, these conformations were added to a solvated cubic box of water molecules using the ‘gmx genion’ script to incorporate various CL ions. The protein was kept within the simulation box at a distance of 1.0 nm from the box’s walls in order to adhere to the minimal image guidelines. This system was brought to equilibrium using NVT and NPT ensembles. It was then subjected to position-restrained MDS for 50 ns, which fixes the backbone C-atoms while permitting solvent molecules to migrate. The covalent contacts were obtained using a linear constraint solver algorithm, and the electrostatic interactions were obtained using the particle mesh Eshwald method [37,38]. Temperature and pressure were adjusted to 300 K and 1 atm, respectively, using the Parrinello-Rahman and the V-rescale weak coupling approach [39]. The leapfrog technique was used to integrate the motion equation at a time step of 2 fs, updating the list of neighbors every 10 steps. Finally, the inbuilt GROMACS scripts were used to produce trajectories of root mean square deviation (RMSD), root mean square fluctuation (RMSF), and hydrogen bond (H bond) graphs from these solvated and equilibrated complexes (three protein–ligand complexes).

### 2.4. MMPBSA Calculations

The final, binding free energies of the simulated protein-ligand complexes were calculated by Molecular Mechanics Poisson Boltzmann Surface Area (MMPBSA) using the gmmpbsa module of gmx MMPBSA [40]. This module is based on an endpoint method for the calculation of binding free energy (BFE) that applies the following equations:

ΔGbinding = ΔH − TΔS

ΔH = ΔE_electrostatic_ + ΔE_vdW_ + ΔG_polar_ + ΔG_nonpolar_

The BFE, in this case, is ΔG_binding_, while the electrostatic contribution is represented by ΔE_electrostatic_, the van der Waals contribution by ΔE_vdW,_ and the polar and non-polar solvation terms, respectively, by ΔG_polar_ and ΔG_nonpolar_. The non-polar solvation term is most frequently referred to as the SASA contribution. To determine the binding energies, MMPBSA combines continuous solvent methods with molecular mechanics and generalized electrostatics [41].

### 2.5. Prediction of ADMET Properties

The absorption, distribution, metabolism, excretion and toxicity (ADMET) properties of the compounds with the highest hits from the molecular docking studies were executed using the webserver Swissadme (http://www.swissadme.ch/index.php#top, accessed on 7 June 2022) [42] and ADMETlab 2.0 (https://admetmesh.scbdd.com/service/, accessed on 7 June 2022) [43]. The hit compounds were accessed for their solubility, pharmacodynamics, and pharmacokinetic properties using the associated models on this web platform.

## 3. Results and Discussion

### 3.1. Molecular Docking of Major Cannabinoids with the RBD-ACE2 Complex of SARS-CoV-2 and SARS-CoV

We used a virtual screen of 42 known cannabinoids to search for potential interaction between cannabinoids and the RBD-ACE2 complex of SARS-CoV-2 and SARS-CoV (Table S1). Molecular docking was used with the AutoDock Vina tool to discover protein-ligand interactions based on affinity characteristics. We docked 42 bioactive cannabis compounds to the active site of the RBD-ACE2 complex, which contained the important binding sites for the SARS-CoV-2 or SARS-CoV peptidase domain [4]. We calculated the total binding energy of all docked bioactive compounds to rank and measure the strength of the protein-ligand interactions. Our findings revealed that luteolin, CBGVA, and CBNA were ranked first, second, and third in our rankings based on their docking score: −6.2, −6.2, −6.3 kcal/mol, respectively (Table S2), and might be considered plausible inhibitors of SARS-CoV-2 RBD-ACE2. While the docking score of luteolin, stigmasterol, and CBNA was −6.8, −7.0, and −6.7 kcal/mol and was thought to be primary inhibitors of SARS-CoV RBD-ACE2. Their docking score was higher than that of luteolin-binding ACE2, −5.23 kcal/mol [15]. Compared to coumarin derivatives [44], the docking score of these three small molecules was all lower but higher than that of other cannabinoids [18]. 

**Figure 1:**
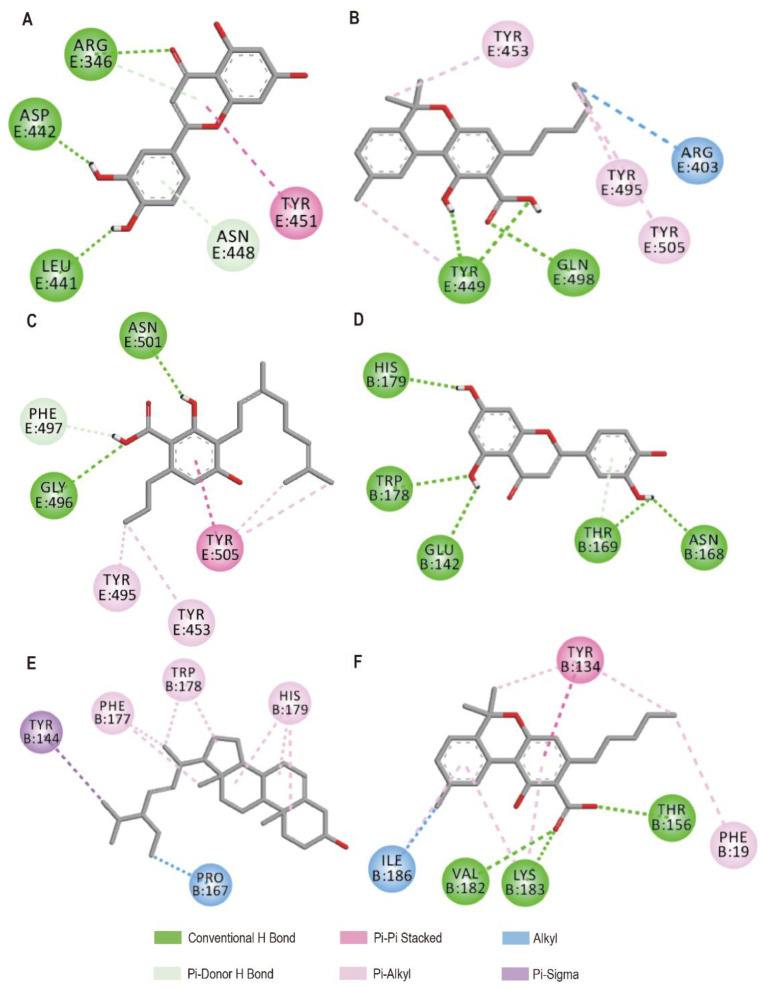
2D interactions of a complex of RBD-ACE2 of SARS-CoV or SARS-CoV-2 with top three bioactive molecules luteolin (**A**), CBNA (**B**), CBGVA (**C**), luteolin (**D**), stigmasterol (**E**), CBNA (**F**).

Then, we thoroughly investigated the interactions of the top three compounds with RBD-ACE2 of SARS-CoV-2 and SARS-CoV. Luteolin interacted with Arg346, Leu441, and Asp442, forming a conventional H bond with each of these three sites, two Pi-Donor H bonds with Arg346 and Asn448, respectively, and one Pi-Alkyl and Pi-Pi bond with Tyr451 (Figure 1A). However, although the molecular structure of luteolin was similar to that of myricetin, myricetin formed a conventional H bond at the His235 and Val292 sites, two pi-pi stacked bonds with Tyr343 and one pi-alkyl bond [21]. The other binding analysis to luteolin with ACE2 revealed 4 H bonds [14]. We discovered that CBNA interacted with Tyr449 and Gln498, producing three conventional H and three pi-alkyl bonds with Tyr453, Tyr495 and Tyr505, and one Alkyl bond with Arg403, respectively (Figure 1B). Similarly, Kuwanon C (KC) was identified that could interact with Gln498 of spike S1 RBD [45]. CBGVA formed two conventional H bonds with Gly496 and Asn501, as well as four pi-alkyl bonds with Tyr453, Tyr495, and Tyr505, which was identical to the KC interaction with ACE2 [45], and one Pi-Pi bond with Tyr505 (Figure 1C). The previous study showed that CBD and CBN formed two and one H bonds, respectively, at the Glu166 and Met165 sites [18]. This differed from that of CBNA; even CBN and CBNA had very similar structures.

Luteolin can interact with five active sites of RBD-ACE2 of the SARS-CoV, Glu142, Asn168, Thr169, Trp178, and His179, and form five regular H bonds and one Pi-Donor H bond (Figure 1D). Similarly, stigmasterol can create seven pi-alkyl bonds and one pi-sigma bond with five active sites: Tyr144, Pro167, Trp178, His179, and Phe177 (Figure 1E). CBNA was found that interact with Phe19, Tyr134, Thr156, Val182, Lys183, Ilp186, and forming three convention H bonds, one alkyl, one pi-pi stacked and six pi-alkyl bonds (Figure 1F). The residues of Arg403, Tyr449, Tyr453, Gln498, Asn501 and Tyr505 were found they can mediate the fusion of SARS-CoV-2 with the cell membrane via the RBD-ACE2 interface [46].

Furthermore, our results revealed that CBNA could bind to Tyr453 of SARS-CoV-2 RBD-ACE2, consistent with the binding between luteolin and ACE2 [16]. This indicates that CBNA and luteolin all have the potential ability to block viral recognition and entry. In contrast, no CBNA and luteolin were found that can bind to Tyr453 of SARS-CoV RBD-ACE2, suggesting that SARS-CoV-2 used a different entry site than SARS-CoV. Although CBD, CBN and CBDA belong to the cannabinoids, the sites and binding ability differ from that of CBNA, and it was suggested that the choice of sites could be critical to SARS-CoV-2control strategies. 

### 3.2. Root Mean Square Deviations

**Figure 2:**
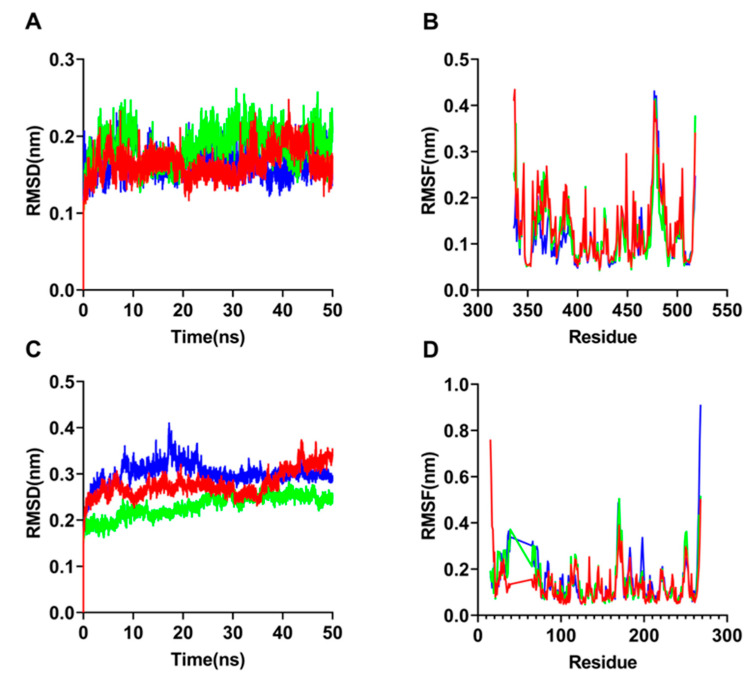
Analysis of RMSD and RMSF on complex of RBD-ACE2 of SARS-CoV or SARS-CoV-2. RMSD analysis on a complex of RBD-ACE2 of SARS-CoV with bioactive molecules: luteolin (red), CBNA (green) and CBGVA (blue) (**A**). RMSF analysis on residues complex of RBD-ACE2 of SARS-CoV towards the bioactive molecules: luteolin (red), CBNA (green) and CBGVA (blue) (**B**). RMSD analysis on a complex of RBD-ACE2 of SARS-CoV-2 with bioactive molecules: luteolin (red), stigmasterol (green) and CBNA (blue) (**C**). RMSF analysis on residues complex of RBD-ACE2 of SARS-CoV-2 towards the bioactive molecules: luteolin (red), stigmasterol (green) and CBNA (blue) (**D**).

MDS is typically regarded as a crucial component of computational analysis due to the entity dynamic of protein-ligand interactions. We used MDS with a time period of 50 ns to evaluate the complex for RMSD, RMSF, and H bonds to gain more insight into the conformational flexibility and stability of ligand molecules. Clusters and MMPBSA analysis were also created to better comprehend complicated interaction paths. Because RMSD is the protein backbone C-α atoms that is one of the indicators of conformational stability, it is one of the main parameters to study complexes of protein-ligand. The estimated findings revealed that RBD-ACE2-luteolin, RBD-ACE2-CBGVA, and RBD-ACE2-CBNA obtained very low magnitude RMSDs for SARS-CoV-2, ranging from 0.1 nm to 0.25 nm (Figure 2A). While the minimum RMSD with a similar structure, myricetin, was 0.2 nm [21], which was less stable than luteolin. This was similar for CBN and CBDA but different from THC and CBD, which showed no additional fluctuation [18]. Significant fluctuations were shown at the active site, with residues receiving significant peaks. This demonstrated that the protein had achieved a high level of conformation stability with the top three ligand molecules. Furthermore, the steady trajectories with consistent and small RMSD plot fluctuations suggested that the protein backbone had minor structural disturbances, despite a few changes at different time points, such as 9 and 41 ns. Similarly, the fluctuations of myricetin occurred at 35 and 42 ns [21]. Furthermore, we estimated the RMSF for protein residues towards these three molecular ligands, and the results revealed considerable changes between active sites and residues ranging from 0.05 to 0.45 nm (Figure 2B). 

The RMSDs for RBD-ACE2 of SARS-CoV were found to be lowest and highest for stigmasterol and CBNA, respectively. These top three molecules have sizes ranging from 0.2 to 0.35 nm and a low magnitude, making them more stable than those of SARS-CoV-2 (Figure 2C). RMSF analysis revealed that the bioactive region with residues had substantially more variations than those of SARS-CoV-2 (Figure 2D). Further evidence that the ligands can fit well in the essential region of the complex can be found in the multiple significant peaks of increased fluctuation that were observed. The measured RMSD indicated that these three small molecules were conformationally stable and did not show large fluctuations. For example, luteolin occasionally exhibited transition conformational fluctuations, such as at 8 and 40 ns, where the RMSD increased from 0.22 to 0.25 nm and then rapidly decreased to about 0.2 nm.

### 3.3. Cluster Analysis

To further illustrate the stability between protein receptor and ligand molecules, we carried out the cluster analysis over the MD trajectories of these complexes for the final 10 ns of the simulation. As shown in Table S3, these three SARS-CoV-2 complexes produced a sizable number of clusters, ranging from 8 to 32, demonstrating the protein’s higher adaptability to ligand molecules and confirming our RMSF analysis. The RMSD measurements were also at astonishingly low levels. At the same time, myricetin generated 49 clusters for SARS-CoV-2 protein-ligand and far more than 32 luteolin [21]. This suggested luteolin was more stable than myricetin due to the larger number of clusters and the higher flexibility of the protein. Contrarily, SARS-CoV had much fewer clusters than SARS-CoV-2, ranging from 10 to 19, but also had lower RMSDs and matrix energies. This was identical to the number of clusters found in Nsp15 of SARS-CoV-2 [21]. This meant that the conformational stability of the clusters was quite low.

### 3.4. Hydrogen Bond and MMPBSA Analysis

**Figure 3:**
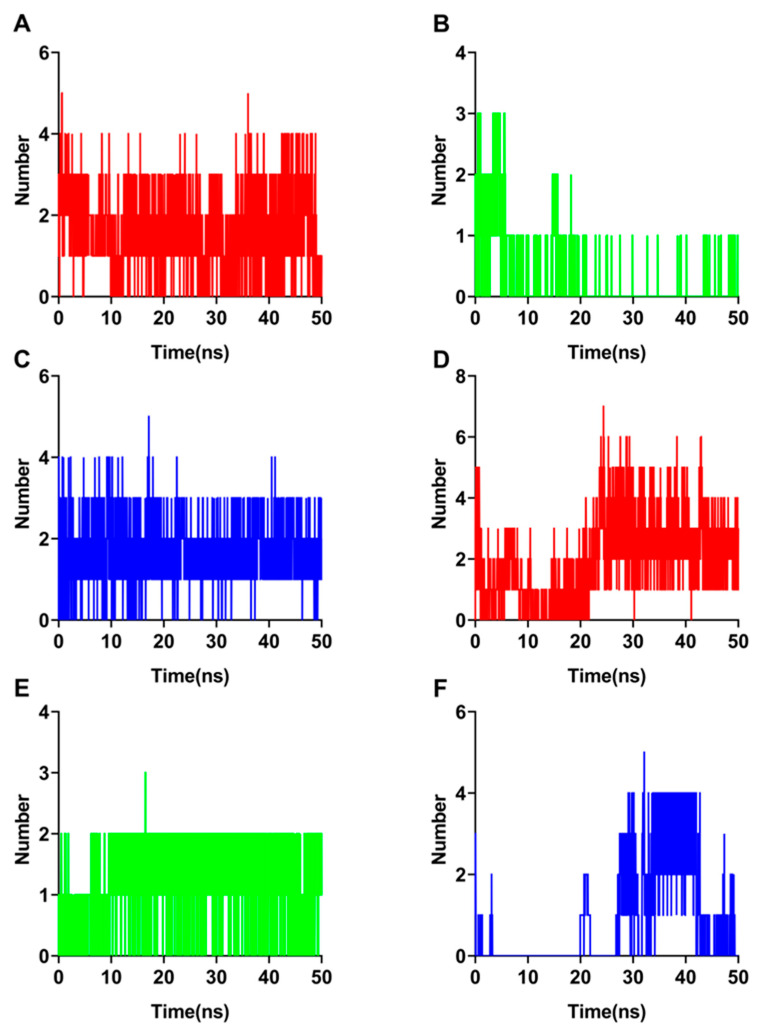
Hydrogen bond profiles of complex RBD-ACE2 of SARS-CoV or SARS-CoV-2. Hydrogen bond profiles of complex RBD-ACE2 of SARS-CoV with bioactive molecules: luteolin (Red) (**A**), CBNA (Blue) (**B**) and CBGVA (Green) (**C**). Hydrogen bond profiles of complex RBD-ACE2 of SARS-CoV-2 with bioactive molecules: luteolin (Red) (**D**), stigmasterol (Blue) (**E**) and CBNA (Green) (**F**).

To further confirm the stable conformation of the protein with three molecular ligands, we performed an H bond analysis. The number of H bonds produced by luteolin with RBD-ACE2 of SARS-CoV-2 ranged from 0 to 5, with considerable fluctuations from time to time (Figure 3A). On the other hand, CBNA produced a high of 3 H bonds with a minimum of 0 H bonds as the simulation went on, while the average number of H bonds declined from 6 ns as the simulation advanced (Figure 3B). In the case of CBGVA, the number of H bonds generated ranged from 0 to 5, with an average of 2 H bonds present throughout the simulation (Figure 3C). Similarly, luteolin established 0 and 7 H bonds with protein at the minimum and maximum for RBD-ACE2 of SARS-CoV, respectively, and then maintained an average of 3 H bonds after 20 ns (Figure 3D). A maximum of 3 H bonds and a minimum of 0 H bonds were generated by stigmasterol (Figure 3E). CBNA, on the other hand, had a maximum of 5 H bonds and a minimum of 0 H bonds (Figure 3F). VMD program analysis showed that the key residues that formed hydrogen bonds interacting between with SARS-CoV-2-RBD and luteolin were Ser349 (60.82%), Asn448 (40.46%), Arg346 (15.12%), Asp442 (10.9%), Asn450 (4.98%) and Leu441 (4.5%). The interactions between SARS-CoV-2-RBD and CBGVA are accompanied by five hydrogen bonds involving Arg403 (64.23%), Gly496 (44.15%), Tyr505 (21.46%), Gln493 (9.54%) and Asn501 (7.48%). While CBNA was found to have four hydrogen bonds with Asn501 (14.6%), Thr500 (3.9%), Gly502 (3.84%) and Gln498 (2.84%). Based on the results of the H bond analysis, the probability of forming H bonds between CBNA and amino acid residues of the protein was significantly lower compared to luteolin and CBGVA, indicating that the stability of CBNA binding to protein was relatively low. In SARS-CoV-RBD, it can form six hydrogen bonds with luteolin: Asn78 (55.31%), Arg257 (28.05%), Thr253 (7.94%), Asp181 (7.84%), Thr82 (7.62%) and Ile175 (6.18%); form six hydrogen bonds with stigmasterol including Asp181 (52.25%), Lys173 (24.5%), Leu174 (9.9%), Thr180 (7.02%),Ile175 (5.3%),His179 (4.92%); form three hydrogen bonds with CBNA: Arg257 (41.79%), Thr82 (27.02%) and Asn145 (4.18%). These three small molecules are highly likely to form H bonds during the molecular simulation process and indicated that all three small molecules are capable of stably binding to the protein. These amino acid active sites can pave the way for directed evolution and rational drug design.

The interaction between protein and ligand was calculated using MMPBSA analysis, which included five parameters: total binding free energy (BFE), van der Waals energy, electrostatic energy, polar solvation energy, and SASA energy. Our findings revealed that CBGVA exhibited the best BFE with RBD-ACE2 of SARS-CoV-2 at −22.9144 kcal/mol, followed by luteolin at −20.7415 kcal/mol and CBNA at −10.9932 kcal/mol (Table S4). The binding energy of CBNA and protein was relatively large, and this was consistent with the probability and number of H bonds formation between CBNA and protein receptors during the simulation. This suggested that CBGVA and luteolin formed a stable complex with the protein receptor, which was validated by the van der Waals energy analysis. For RBD-ACE2 of SARS-CoV, stigmasterol had the lowest BFE (−28.0828 kcal/mol), followed by CBNA (−17.0425 kcal/mol) and luteolin (−12.7945 kcal/mol). The binding energy of these three small molecule ligands with the protein receptor is low, which was consistent with the results that all three small molecules have a higher probability of forming H bonds with the protein-ligand. Raj et al. [18] found that CBN can effectively inhibit the M^pro^ of SARS-CoV-2, with a binding affinity of −10.42 kcal/mol, while our CBNA can inhibit the RBD-ACE2 complex with a binding affinity of-17.68 kcal/mol. The binding energy of luteolin with M^pro^ and ACE2 was −8.2 and −10.1 kcal/mol, respectively [14]. Although KC has the ability to block RBD-ACE2 receptor interaction, the binding energy was −7.1 kcal/mol [45]. This suggests that different cannabis target proteins are different on SARS-CoV-2. When luteolin was targeted at RdRP by molecular docking, and its docking score was −7.62 kcal/mol [15], it suggested there were differences in inhibition of SARS-CoV-2 even when the same bioactive molecule was targeted on different proteins. The binding energy of luteolin-inhibited cell entry was −36.82 kJ/mol [16], while CBDA was −6.3 kcal/mol [17]. 

### 3.5. A Potential Mechanism of Cannabinoids Inhibited SARS-CoV-2 and SARS-CoV

Based on the molecular docking, RMSD, cluster and MMPBSA results, this indicated that luteolin and CBNA could stably bind to regions of RBD-ACE2 of SARS-CoV-2 or SARS-CoV. Furthermore, luteolin and CBNA may inhibit SARS-CoV-2 or SARS-CoV by binding to the RBD regions, preventing virus entry and slowing down viral disease progression (Figure S1). The binding between the S-protein of SARS and ACE2 (cellular receptor) initiates the SARS life cycle in host lung cells and forms a critical area known as the RBD. According to a prior study, CBD inhibited the M^pro^ of SARS-CoV-2 and reduced the translation and viral genome release [21]. In addition, it was confirmed luteolin could efficiently inhibit M^pro^ of SARS-CoV-2 in a vitro experiment [47]. Our findings revealed that luteolin and CBNA, two other cannabinoids, may prevent the entry of SARS-CoV-2 or SARS-CoV. These two cannabinoids can bind to RBD in the pathway and prevent viral entrance. Meanwhile, they can interact with ACE2 to cause immunosuppression and decrease the generation of inflammatory cytokines based on previous studies [16,48,49].

### 3.6. ADMET Properties Analysis

The absorption, distribution, metabolism, excretion, and toxicity profiles of the three docked molecules of the RBD-ACE2 complex of SARS-CoV-2 or SARS-CoV were checked using 45 classification models utilizing Swissadme and Admetlab 2.0 server (Table S5). The molecular weights of these three small molecules were less than 500 and within an acceptable range, indicating that they are likely to exhibit good absorption and pharmacological activity. In particular, lutein and CBNA have high absorption and bioavailability and, therefore, could be considered effective therapeutic candidates against SARS-CoV-2 or SARS-CoV. It also predicts the isoform of the five cytochromes (CYP) that play a critical role in drug clearance (Table S6). Although the clearance level of CBNA and stigmasterol was lower than that of luteolin, they were all within the acceptable range. The level of excretion of luteolin, CBNA and stigmasterol was a big difference. For example, the CL of luteolin, CBNA and stigmasterol were 8.146, 1.072 and 4.515, respectively (Table S6). Luteolin inhibited CYP1A2, CYP2D6, and CYP3A4, while CBNA inhibited CYP1A2, CYP2C19, and CYP2C9, implying that luteolin and CBNA inhibit liver metabolism. None of them could cross the blood-brain barrier, and none of the substances would be metabolized by any of the inhibitors evaluated for drug metabolism. 

A variety of toxicities have been assessed for these three molecules, including those affecting human health and the environment. Three cannabinoids were evaluated using ADMET profiling and found to be acceptable and safe. These findings indicate that all of the ligands had no significant toxicity issues such as carcinogenic, tumorigenic, or adverse reproductive health effects, implying that they are relatively safe for oral administration (Table S6). Luteolin can inhibit hERG and has been predicted to be a skin sensitization, suggesting it potentially causes side effects. Environmental toxicity showed that these three small molecules caused negligible toxic effects. As shown in Table S6, no acute oral toxicity and very slight bioconcentration factors such as luteolin 1.016 were found.

## 4. Conclusions

Viral entry was crucial to the invasion of the host cell. In a recent investigation, luteolin and CBNA were found to have antiviral properties against SARS-CoV-2 and SARS-CoV. It can be concluded that luteolin and CBNA not only restrict virus entry by blocking the RBD-ACE2 complex, which was previously thought to be responsible for membrane fusion but also modulates the immune system, as other cannabinoids such as CBD have demonstrated [17]. The top three bioactive substances were strongly associated with the main viral entrance sites, according to our research, indicating that they could be used as a potential inhibitor against severe acute respiratory syndrome. Thus, luteolin and CBNA can be a potential inhibitor to avoid COVID-19 or severe acute respiratory syndrome, although their inhibitory effects in vivo and in vitro need to be investigated further.

## Data Availability

Not applicable.

## Supplementary Materials

The following supporting information can be downloaded at: https://www.mdpi.com/article/10.3390/biom12121729/s1, Figure S1: A possible mechanism of cannabinoids to inhibit SARS-CoV or SARS-CoV-2. SARS-CoV or SARS-CoV-2 life cycle in host lung cells is initiated through forming a complex of RBD-ACE2 by binding between viral spike glycoprotein and ACE2 cellular receptor, and then these cannabinoids will block the formation of the complex of RBD-ACE2 and progress to inhibit the entry of the virus. Table S1: Bioactive moleculars; Table S2: Docking score for cannabis compounds; Table S3: The number of clusters formed for a protein-ligand complex, their average RMSD and the energy of matrix; Table S4: MMPBSA based on total binding free energies along with its constituent energies for top three bioactive molecules; Table S5: The ADME profiling enlisting absorption and metabolism parameters of best-selected molecules; Table S6: The representative ADMET profiling enlisting absorption, metabolism, excretion and toxicity-related parameters of best-selected molecules.
