# Supplementary material for: Analysis and Identification of Bioactive Compounds of Cannabinoids in Silico for Inhibition of SARS-CoV-2 and SARS-CoV
Source: Biomolecules. 2022 Nov 22;12(12):1729. doi: 10.3390/biom12121729 (PMC9775500; doi:10.3390/biom12121729)
Supplement: Supplementary file 1 [file biomolecules-12-01729-s001.zip › Table S3 The number of clusters formed for a protein.pdf]

Table S3 The number of clusters formed for a protein-ligand complex, their average RMSD and the energy of matrix.

Protein	Complex	Number of clusters	Average RMSD	Energy of matrix
6M17	Luteolin	32	0.141506	5.66384
6M17	CBGVA	31	0.139141	5.54774
6M17	CBNA	8	0.102055	5.14521
3R4D	Luteolin	19	0.129989	4.76544
3R4D	Stigmasterol	10	0.113522	4.49758
3R4D	CBNA	10	0.112141	4.58328
