# Supplementary material for: Analysis and Identification of Bioactive Compounds of Cannabinoids in Silico for Inhibition of SARS-CoV-2 and SARS-CoV
Source: Biomolecules. 2022 Nov 22;12(12):1729. doi: 10.3390/biom12121729 (PMC9775500; doi:10.3390/biom12121729)
Supplement: Supplementary file 1 [file biomolecules-12-01729-s001.zip › Table S4 MMPBSA.pdf]

Table S4 MMPBSA based on total binding free energies along with its constituent energies for top three bioactive molecules.

Protein	Complex	Total Binding Free Energy (Kcal/mol)	Van der Waals Energy (Kcal/mol)	Electrostatic Energy (Kcal/mol)	Polar Solvation Energy (Kcal/mol)	SASA Energy (Kcal/mol)
6M17	Luteolin	-20.7415	-24.6780	-15.8804	23.1922	-3.3753
6M17	CBGVA	-22.9144	-26.5570	-18.3969	25.5149	-3.4755
6M17	CBNA	-10.9932	-15.0076	-7.6496	13.7080	-2.0440
3R4D	Luteolin	-12.7945	-18.2797	-22.3411	30.6544	-2.8280
3R4D	Stigmasterol	-28.0828	-35.6418	-6.9927	18.7811	-4.2294
3R4D	CBNA	-17.0425	-18.6689	-22.9830	27.2120	-2.6027
