# Supplementary material for: Analysis and Identification of Bioactive Compounds of Cannabinoids in Silico for Inhibition of SARS-CoV-2 and SARS-CoV
Source: Biomolecules. 2022 Nov 22;12(12):1729. doi: 10.3390/biom12121729 (PMC9775500; doi:10.3390/biom12121729)
Supplement: Supplementary file 1 [file biomolecules-12-01729-s001.zip › Table S6.pdf]

Table S6 The ADMET profiling enlisting absorption, metabolism, and toxicity-related parameters of best selected molecules.

**Molecule**	**Formula**	**MW**	**Log s**	**Abs**	**BBB**	**Pgp-s**	**CYP1A2**	**CYP2C19**	**CYP2C9**	**CYP2D6**	**CYP3A4**	**Bio**	**AOT**	**CL**	**T1/2**	**hERG blocker**	**Oral acute toxicity**	**Bioconcentration factors**
Luteolin	C15H10O6	286.2	-3.71	High	No	No	Yes	No	No	Yes	Yes	0.55	-	8.146	0.898	NO	NO	1.016
CBNA	C22H26O4	354.4	-5.95	High	No	No	Yes	Yes	Yes	No	No	0.85	-	1.072	0.164	NO	NO	0.426
Stigamasterol	C29H48O	412.6	-7.46	Low	No	No	No	No	Yes	No	No	0.55	-	4.515	0.036	NO	NO	3.047
