# Supplementary figures and images for: Analysis and Identification of Bioactive Compounds of Cannabinoids in Silico for Inhibition of SARS-CoV-2 and SARS-CoV
Source: Biomolecules. 2022 Nov 22;12(12):1729. doi: 10.3390/biom12121729 (PMC9775500; doi:10.3390/biom12121729)

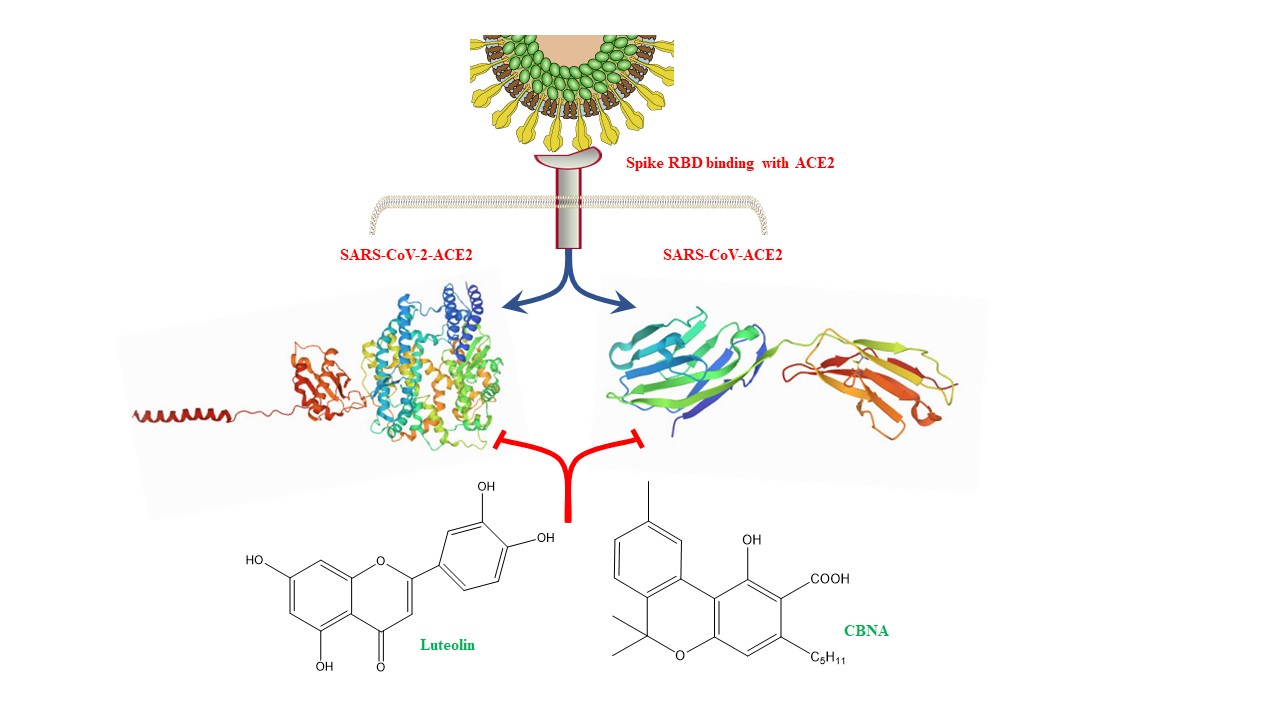

Supplement: Supplementary file 1 [file biomolecules-12-01729-s001.zip › Figure S1.jpg]
